# An Albumin-Binding Domain Peptide Confers Enhanced Immunoprotection Against Viral Myocarditis by CVB3 VP1 Vaccine

**DOI:** 10.3389/fimmu.2021.666594

**Published:** 2021-09-22

**Authors:** Yue Gao, Yan Yue, Sidong Xiong

**Affiliations:** Jiangsu Provincial Key Laboratory of Infection and Immunity, Institutes of Biology and Medical Science, Soochow University, Suzhou, China

**Keywords:** Coxsackievirus B3, viral myocarditis, ABD, draining lymph nodes, vaccine, CTL

## Abstract

*Coxsackievirus B3 (CVB3)*-induced viral myocarditis is a common clinical cardiovascular disease without effective available vaccine. In this study, we tried to potentiate the immunoprotection efficacy of our previous *CVB3*-specific VP1 protein vaccine by introducing a streptococcal protein G-derived, draining lymph nodes (dLNs)-targeting albumin-binding domain (ABD) peptide. We found that compared with the original VP1 vaccine, ABD-fused VP1 (ABD-VP1) vaccine gained the new ability to efficiently bind murine albumin both *in vitro* and *in vivo*, possessed a much longer serum half-life in serum and exhibited more abundance in the dLNs after immunization. Accordingly, ABD-VP1 immunization not only significantly facilitated the enrichment and maturation of dendritic cells (DCs), induced higher percentages of IFN*-γ^+^* CD8*^+^* cells in the dLNs, but also robustly promoted VP1-induced T cell proliferation and cytotoxic T lymphocyte (CTL) responses in the spleens. More importantly, ABD-VP1 also elicited higher percentages of protective CD44^hi^ CD62L^hi^ memory T cells in dLNs and spleens. Consequently, obvious protective effect against viral myocarditis was conferred by ABD-VP1 vaccine compared to the VP1 vaccine, reflected by the less body weight loss, improved cardiac function, alleviated cardiac histomorphological changes and an increased 28-day survival rate. Our results indicated that the ABD might be a promising immune-enhancing regime for vaccine design and development.

## Introduction

Viral myocarditis (VMC) is a kind of common cardiovascular disease (1), and represents the main cause of sudden cardiac death in young adults under 40 years of age (2–5). *Coxsackievirus B3 (CVB3)* is considered as one of the common etiological agents of VMC (5–7).

Despite the tremendous progress in elucidating the viral structure, protein function and viral pathogenesis of *CVB3*, so far no prophylactic vaccine is available in clinic. *CVB3* capsid protein VP1 is an immune-dominant antigen and rich of T/B cell epitopes (8). Compared with other structural proteins, VP1 immunization could induce stronger *CVB3*-specific cellular and humoral immune responses (9), therefore it usually served as a promising vaccine candidate. Our previous studies have shown that inoculation of VP1 vaccine can elicit certain protective *CVB3*-specific responses (10), but its immunoprotection efficacy needs to be further improved.

Rapid and effective enrichment of antigens in dLNs is a key step for vaccines to successfully elicit immune responses (11, 12). Targeting dLNs not only greatly increases the local antigen deposition, but also facilitates the antigen uptaking and processing by antigen-presenting cells (11). Both of them are critical for the potent immunoprotection induction. In addition, delivering vaccine to dLNs can more obviously enhance immune responses compared with the peripheral vaccination (13). Therefore, targeting-dLNs has gradually become an important vaccine-enhancing strategy, and receives more and more attention. Among them, one approach utilizes the high enrichment of serum albumin in the dLNs.

Albumin is a protein abundant in plasma and lymph, occupied about 50%-60% of total protein in plasma (14). Initially, albumin was used to track cancer metastasis by binding a contrast agent or dye that locates lymph node aggregation (15). Later, “albumin hitchhiking” effect was used by more small molecule compounds and peptide drugs to achieve dLNs enrichment (16). Meanwhile, coupling with long half-life albumins would also obviously increase the *in vivo* retention periods of oligonucleotides (17), compounds (18) or proteins (19).

Herein, we were concerned with the albumin-binding domain (ABD) derived from the streptococcal protein G. ABD is a 46-residue peptides, which has a very high binding affinity with human, monkey and mouse albumins (20). Previous studies showed that ABD could efficiently direct the coupled substances to dLNs, and could increase their retention times and stabilities *in vivo (* 21). While so far, limited number of studies reported the effect of ABD-based dLNs-targeting approaches on vaccine efficacy.

In this study, we introduced ABD peptide into our previously prepared VP1 protein vaccine, and evaluated the elicited immune responses and protective effect of this novel ABD-VP1 vaccine. We found that it efficiently bound the mouse serum albumin (MSA) and exhibited a long half-life *in vivo*. Compared with VP1 vaccine, ABD-VP1 was more abundantly enriched in dLNs, and could efficiently induce higher percentages of IFN*-γ^+^* cells in both dLNs and spleens. Meanwhile, it also induced a higher level of *CVB3*-specific neutralizing antibodies in serum. More importantly, ABD-VP1 also elicited higher percentages of protective CD44^hi^ CD62L^hi^ memory T cells in dLNs and spleens. These enhanced immune responses led to an improved effect of protection against viral myocarditis, evidenced by alleviated myocardial inflammation, and reduced cardiac viral loads in immunized mice after *CVB3* challenge.

## Materials and Methods

### Cells and Virus

HeLa cells (ATCC catalog number: CCL-2) were cultured in Dulbecco’s modified Eagle’s medium (DMEM, Hyclone). SP2/0 cells (Mouse myeloma cells) were cultured in RPMI-1640 medium (Hyclone), both medium supplemented with 10% fetal bovine serum (FBS, Hyclone), 2 mM L-glutamine, and 1% penicillin/streptomycin (Invitrogen). Cells were cultured in a 5% CO_2_ incubator at 37°C. *CVB3* virus (Nancy strain) amplification was achieved by infecting HeLa cells.

### Mice Immunization

Male BALB/c (H-2^d^) mice, 6-8weeks of age, were purchased from the Experimental Animal Center of the Chinese Academy of Sciences (Shanghai, China), and were bred and maintained in a SPF-level laboratory animal room. Mice were subcutaneously immunized with 25 μg (in 100 µl) ABD-VP1 or VP1 protein at the tail bases 3 times with 2-week intervals. All animal experiments were performed in accordance with the guidelines for the Care and Use of Laboratory Animals (Ministry of Health, China, 1998). All animal experiment protocols in this study were approved by the Ethics Committee of Soochow University. At the end of experiments, mice were euthanized by cervical dislocation.

### Preparation of Recombinant ABD-VP1 and VP1 Vaccines

The ABD gene was cloned into the previously prepared pET28a-VP1 plasmid through BamH I digestion sites. For evaluating dLNs accumulation of ABD-VP1 or VP1, the mCherry gene was cloned into the pET28a-ABD-VP1 or pET28a-VP1 plasmid. The inserted fragments were verified by DNA sequencing (Genewiz). Recombinant proteins were produced by the E.coli prokaryotic expression system and purified with His-beads. The specific expression and purification of those recombinant proteins were conducted as previously reported (22). Purified proteins were confirmed by western blot assays with the anti-VP1 antibody (Dako).

### ELISA Assays

To analyze the albumin-binding affinity of ABD-VP1 vaccine, ELISA plates were coated with bovine serum albumin (BSA) or MSA (10 μg/ml, Sigma) overnight at 4°C, and blocked with 5% BSA for 1 h at room temperature (RT). After incubating with VP1 or ABD-VP1 protein (1 μg/ml) for 2 h at RT, plates were incubated with anti-VP1(1:8000) and HRP-labeled goat anti-mouse secondary antibodies (1:6000, Southern Bio) sequentially at RT for 1 h respectively. The reaction was stopped by adding 2 M H_2_SO_4_, and the absorbance at 450 nm was measured with a microplated reader (BioTeK).

To determine the serum half-lives of ABD-VP1 and VP1 proteins, mice were given 25 μg ABD-VP1 or VP1 protein in a volume of 100 μl. Sera were extracted from the blood taken *via* mandibular vein puncture at indicated time points and incubated with the VP1 protein pre-coated ELISA plates for 1h at RT. Then the plates were incubated with anti-VP1 and HRP-labeled goat anti-mouse secondary antibody sequentially at RT for 1 h respectively. The reaction was stopped and the absorbance at 450 nm was measured finally.

To detect whether ABD-VP1 could form complex with serum albumin *in vivo*, serum of ABD-VP1- or VP1-immunized mice was added to the ELISA plates which were pre-coated with anti-VP1 antibody (Dako) and then anti-albumin antibody (Proteintech) and HRP-labeled anti-rabbit secondary antibodies were added sequentially and incubate at RT for 1h respectively. Finally, the reaction was stopped, and the absorbance at 450 nm was measured.

### Neutralizing Antibody Titers

Sera were extracted from the blood *via* mandibular vein puncture 1 week after the final immunization, and the levels of *CVB3*-specific neutralizing antibodies were detected as previously described (23).

### CTL Responses and T Cell Proliferation Assays

One week after the final immunization, splenocytes from immunized mice were prepared and subjected to the T cell proliferation assays with a BrdU ELISA (Roche) kit as previously described (24). Briefly, splenocytes (4×10^4^/ells/well) were cultured in 96-well plates for 48 h, and then BrdU labelling solution added according to the manufacturer’s instructions and incubated for 24 h. The reaction was terminated by adding stop solution, and the absorbance at 450 nm was measured with a microplated reader (BioTeK).

To determine the *CVB3*-spcific CTL responses induced by ABD-VP1 or VP1 vaccine, a lactate dehydrogenase (LDH) assay kit was used (Roche). Briefly, VP1 protein-stimulated SP2/0 cells were used as the target cells (1×10^4^ cells). Splenocytes stimulated with 10 μg/ml VP1 protein *in vitro* for 24 h were used as effector cells, and incubated with target cells for 6 h. Then cell supernatant (50 μl/well) was removed and transferred into the corresponding wells of a 96-well plate. Reaction mixture (50 μl) was added to each well and incubated for 30 min at RT. The absorbance of the samples at 450 nm was measured.

The percentage cytotoxicity of CTL was calculated as follows: Cytotoxicity (%) = (effector and target cell mix–effector cell control)–low control/(high control–low Control) ×100%. Low control: the LDH activity released from the untreated normal cells (spontaneous LDH release). High control: the LDH activity released from the lysed cells, provided as the positive control in LDH assay kits (maximum LDH release).

### Flow Cytometry Analysis

One week after the final immunization, splenocytes and dLNs (inguinal lymph nodes) were collected, co-cultured with 10 μg/ml VP1 protein for 24 h, and then stimulated with PMA (50 ng/ml, Sigma-Aldrich), Ionomycin (1 μg/ml, Sigma-Aldrich) and Brefeldin A (eBioscience) for another 6 h before subjected to intracellular IFN*-γ* detection. Subsequently, Cells were stained with peridinin-chlorophyll-protein (PerCP)-conjugated anti-CD4 (BD Biosciences) or allophycocyanin (APC)-conjugated anti-CD8 antibody (BD Biosciences). After fixation and permeabilization using Cytofix/Cytoperm™, cells were stained with PE-conjugated anti-IFN*-γ* antibody (BD Biosciences). To detect the recruitment and maturation of dendritic cells (DCs), cells were stained with PE-conjugated anti-CD11c (BD Biosciences), FITC-conjugated anti-MHCII (BD Biosciences), PerCP-conjugated anti-CD80 (BD Biosciences) and APC-conjugated anti-CD86 antibodies (BD Biosciences). For detection of memory T cells, cells of spleens and dLNs were isolated 8 weeks after the final immunization, and subjected to cell surface staining with APC-conjugated CD3 (Biolegend), FITC-conjugated CD44 (Biolegend), and PE-conjugated CD62L (Biolegend). Then subjected to the flow cytometry assays with a BD FACS Canto™II instrument. Isotype controls and fluorescence minus one (FMO) controls were performed in parallel. And subsequent gating was determined by FMO and isotype controls. All data were analyzed using FlowJo software.

### *CVB3* Challenge

Two weeks after the final immunization, mice were intraperitoneally infected with 3 × 50% lethal dose (LD_50_) *CVB3*. Body weights of mice were taken as initial body weight at the day of virus challenge. Seven days later, mice were weighed again, and the body weight loss rate was calculated as following: [1-(body weight at day 7 post challenge/body weight at the day of challenge)] x 100%. For survival rate, a lethal dose of *CVB3* (5LD_50_) was administrated 2 weeks following the final immunization and survival rate was monitored until day 28 post infection.

### Histomorphological Analysis

Hearts were harvested 7 days after 3LD_50_ *CVB3* challenge, fixed in 10% phosphate-buffered formalin, embedded with paraffin and sectioned followed by the hematoxylin and eosin (HE) staining. Histomorphological analysis was performed on the left ventricle with an aid of a microscope eyepiece grid as previously described (25–28). The inflammation percentages were calculated as following: [the number of intersections on the myocytes with inflammatory cells/the total number of intersections on the myocytes with and without inflammatory cells] ×100%. Sections were scored in a blinded manner by two investigators separately.

### Echocardiography

Seven days after 3LD_50_ *CVB3* challenge, mice were anesthetized with isoflurane by a Vevo compact anesthesia system. The left ventricular ejection fraction (LVEF) and left ventricular fractional shortening (LVFS) were assessed with an echocardiography system (Vevo2100, Visual Sonics, Canada) according to the operator’s manual.

### Adoptive Transfer of Memory T Cells

Splenocytes and dLNs of ABD-VP1 immunized mice were prepared 8 weeks after the final immunization, and CD44^hi^ CD62L^hi^ memory T cells were sorted with a BD FACS Arial III instrument. Naive mice were received memory T cells (3x10^5^/mice) by intravenous injection at the day of *CVB3* infection (3LD_50_). And 7 days later, the histomorphological changes of hearts were evaluated in the recipient mice.

### Statistical Analysis

Statistical analyses were performed using GraphPad Prism 5.01 Software (La Jolla, CA) and presented as the means ± SEM. All statistical analysis was done by using One-Way ANOVA followed by Tukey’s *post hoc* test. The Anderson−Darling test was used to evaluate normality before statistical tests. Survival rate was calculated by Kaplan–Meier analysis. The statistical significance level was set as *P < 0.05; **P < 0.01; ***P < 0.001.

## Results

### ABD Peptide Endowed VP1 Vaccine With a Good Albumin-Binding Ability and an Increased *In Vivo* Half-Life

**Figure 1:**
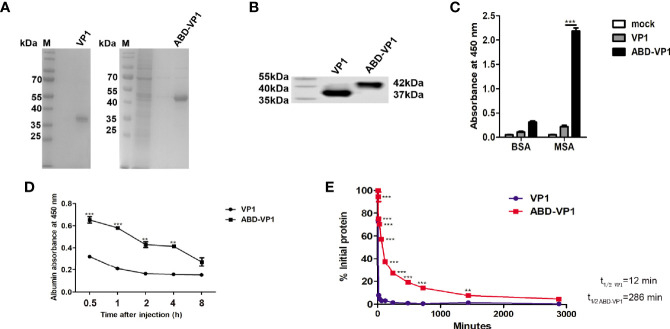
Albumin-binding ability of ABD-VP1vaccine. **(A)** SDS-PAGE and **(B)** western blot analysis of the recombinant ABD-VP1 and VP1 proteins. **(C)** The binding abilities of ABD-VP1 and VP1 proteins to albumins were detected by ELISA assays. **(D)** Serum complex of ABD-VP1 with albumin was detected by ELISA assays. **(E)** The serum half-lives of ABD-VP1 and VP1 vaccines. VP1 and ABD-VP1 proteins were injected subcutaneously into mice, sera were taken at indicated time points for VP1 amount detection by ELISA assays. The level of serum VP1 protein at 5 min after injection was uniformly quantified as 100%. Individual experiment was repeated 3 times. *P < 0.05; **P < 0.01; ***P < 0.001.

Recombinant VP1 and ABD-VP1 proteins were produced by the E.coli prokaryotic expression system and purified with His-beads. The protein purity, integrity and specificity were analyzed by SDS-PAGE (**Figure 1A**) and western blot assays (**Figure 1B**). As expected, ABD conferred VP1 protein with the binding ability to MSA, but not BSA (**Figure 1C**). This excluded the possibility of nonspecific binding of ABD-VP1 protein with the MSA.

To further evaluate the albumin-binding ability of ABD-VP1 *in vivo*, mice were injected subcutaneously with VP1 or ABD-VP1 protein, and 0.5 h later, sera were collected and subjected to the sandwich ELISA assays. As shown in **Figure 1D**, the serum OD450 value of ABD-VP1-treated mice achieved about 0.7 at as early as 1h, and significantly higher than the control VP1 group at all detected time points except for 8 h, indicating that ABD-VP1 efficiently formed complex with the MSA *in vivo*.

It was also found that the half-life of ABD-VP1 was substantially prolonged and achieved as long as about 280 min in the murine serum, which was in sharp contrast to the less than the 15 min half-life of VP1 vaccine (**Figure 1E**), indicating that ABD successfully endowed VP1 with an obviously extended *in vivo* half-life.

### ABD-VP1 Vaccine Efficiently Enriched in the dLNs Post Subcutaneous Immunization

**Figure 2:**
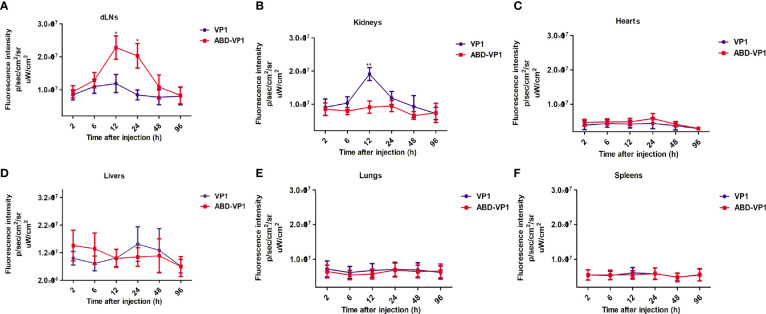
The bio-distributions of ABD-VP1 and VP1 vaccines. Mice were subcutaneously given mCherry-fused ABD-VP1 or mCherry-fused VP1 vaccine, and the vaccine distributions in **(A)** dLNs, **(B)** kidneys, **(C)** hearts, **(D)** livers, **(E)** lungs, **(F)** spleens were analyzed by the bioluminescence imaging assays at the indicated time points. Each group contained 6 mice. *P < 0.05; **P < 0.01; ***P < 0.001.

To observe the *in vivo* distribution, red fluorescence protein mCherry-fused VP1 or ABD-VP1 protein was subcutaneously injected at the tail bases of mice. Tissues of lungs, kidneys, spleens, hearts, livers and dLNs were collected at indicated time points and imaged with the *in vivo* imaging system (IVIS). The results in **Figure 2** showed that ABD-VP1 protein began to obviously enrich in the dLNs at 6 h post inoculation, and achieved the peak at 12 h and persisted at a higher level after 24 hours (**Figure 2A**). As to the VP1 protein, except of a transient and slight enrichment in the dLNs at 12h, hardly significant increases was observed during the whole observation period. Besides, comparable bio-distributions of these two vaccines were evidenced in hearts, livers, lungs, spleens and kidneys, apart from a transient and significant accumulation of the VP1 vaccine in kidneys at 12 h (**Figures 2B–F**). It seemed that ABD-VP1 might less easily be excreted from kidneys. This also coincided with the longer serum retention time of ABD-VP1 vaccine.

### ABD-VP1 Immunization Induced High Percentages of IFN*-γ^+^* CD8*^+^* Cells in dLNs by Promoting the Recruitment and Maturation of Dendritic Cells

**Figure 3:**
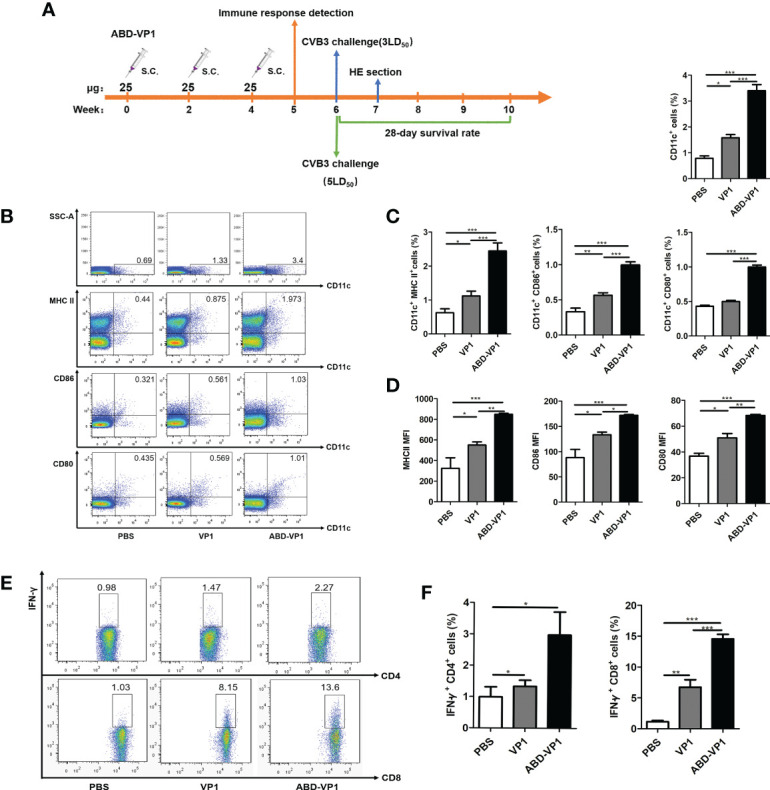
ABD-VP1-induced local immune responses in the dLNs. **(A)** Schematic diagram of the immunization procedure. **(B)** FACS analysis of DC recruitment and maturation in the dLNs of immunized mice. **(C)** Statistical analysis of **(B)**. **(D)** Mean fluorescence intensity (MFI) of MHC II, CD86 or CD80 molecule on CD11c^+^ DC in the dLNs. **(E)** FACS analysis of IFN*-γ^+^* CD8*^+^* and IFN*-γ^+^* CD4*^+^* cells in the dLNs (unstimulated background has been subtracted). **(F)** Statistical analysis of **(E)**. Each group contained 8 mice. *P < 0.05; **P < 0.01; ***P < 0.001.

The immunization and challenge procedures were carried out according to **Figures 3A**. As shown in **Figures 3B, C**, the percentage of CD11c^+^ DCs in ABD-VP1-immunized mice was up to 3.40%, significantly higher than 1.58% of VP1 group, P<0.001. Meanwhile, obviously increased frequency and mean fluorescence intensity (MFI) of MHC II, CD80 or CD86 on CD11c^+^ cells were also evidenced in ABD-VP1 group (**Figure 3D**). Accordingly, the percentage of IFN*-γ* ^+^ CD8*^+^* cells in ABD-VP1 group was significantly higher than that of VP1 group (14.55% *vs* 6.72%, P<0.001, **Figures 3E, F**). In terms of IFN*-γ* ^+^ CD4*^+^* cell percentages, a slight but not significant increase was evidenced in ABD-VP1 group compared with VP1 group. These data clearly indicated that ABD-mediated more abundant accumulation of VP1 in dLNs facilitated the recruitment and maturation of DCs, and promoted the IFN*-γ^+^* CD8^+^ cell responses.

### ABD-VP1 Immunization Enhanced the Splenic T Cell Immune Responses in Mice

**Figure 4:**
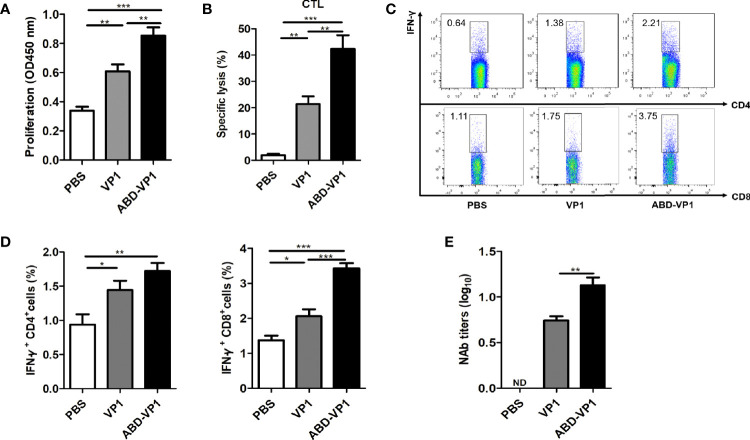
ABD-VP1-induced splenic T cell immune responses. **(A)** *CVB3*-specific T cell proliferation in the spleens of immunized mice. **(B)** *CVB3*-specific CTL activity in the spleens. **(C)** Percentages of IFN*-γ^+^* CD8*^+^* and IFN*-γ^+^* CD4*^+^* cells in the spleens (unstimulated background has been subtracted). **(D)** Statistical analysis of **(C)**. **(E)** Levels of serum neutralizing antibody in immunized mice. Each group contained 6 mice. *P < 0.05; **P < 0.01; ***P < 0.001.

To detect *CVB3*-specific systemic T cell immune responses, murine splenocytes were prepared 1 week after the final immunization and subjected to T cell proliferation, CTL and intracellular IFN*-γ^+^* flow cytometry assays. It was found that ABD-VP1 induced a significantly stronger *CVB3*-specific T cell proliferation response and a higher CTL specific lysis response than VP1 vaccine (**Figures 4A, B**). In addition, the percentage of IFN-*γ^+^* CD8^+^ cells in ABD-VP1 group was 3.42%, significantly higher than the 2.06% of VP1 group; while comparable percentages of IFN*-γ^+^* CD4^+^ were evidenced in these two vaccines (**Figures 4C, D**). The above results indicated that the introduction of ABD could potently enhance systemic CTL and IFN-*γ* ^+^ CD8^+^ cell immune responses elicited by VP1 vaccine. Meanwhile, we also noticed the significant higher neutralizing antibodies in ABD-VP1 immunized mice compared with the VP1 group (**Figure 4E**).

### ABD-VP1 Vaccine Efficiently Protected Mice Against *CVB3* Challenge

**Figure 5:**
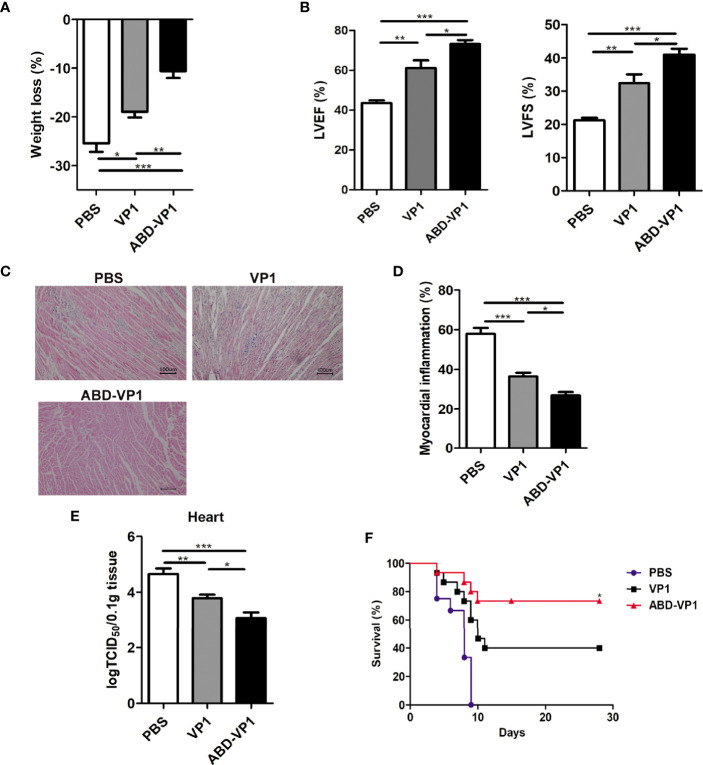
Enhanced immunoprotection against viral myocarditis induced by ABD-VP1 vaccine. Mice were challenged with 3LD_50_ *CVB3* 2 weeks post the final immunization, and myocarditis severity was evaluated 7 days later by **(A)** body weight loss, **(B)** cardiac function and **(C)** histomorphological changes of heart tissues. **(D)** Statistical analysis of **(C)**. **(E)** Meanwhile, viral loads in the hearts were also detected. **(F)** A 28-day survival rate was observed after 5LD_50_ *CVB3* infection. *P < 0.05 for VP1 versus ABD-VP1. Each group contained 8 mice. For histomorphological observation, each group contained 6 mice. For survival rate evaluation, each group contained 15 mice. *P < 0.05; **P < 0.01; ***P < 0.001.

Two weeks after the final immunization, mice were challenged with 3LD_50_ *CVB3*, and the myocarditis severity was assessed by weight loss and cardiac histomorphological observation 7 days later. It was found that compared with VP1 vaccine, ABD-VP1 immunization led to the smaller body weight loss (**Figure 5A**), improved cardiac functions reflected by the increased LVEF and LVFS (P<0.05, **Figure 5B**), milder myocardial inflammatory infiltration and necrosis (**Figures 5C, D**), and an increased 28-day survival rate from about 40% to 73% (**Figure 5F**). This enhanced immunoprotection of ABD-VP1 vaccine was attributed to its more efficient viral control in the hearts (**Figure 5E**).

### Adoptive Transfer of Memory T Cells Derived From ABD-VP1-Immunized Mice Robustly Protected Naive Mice Against *CVB3* Challenge

**Figure 6:**
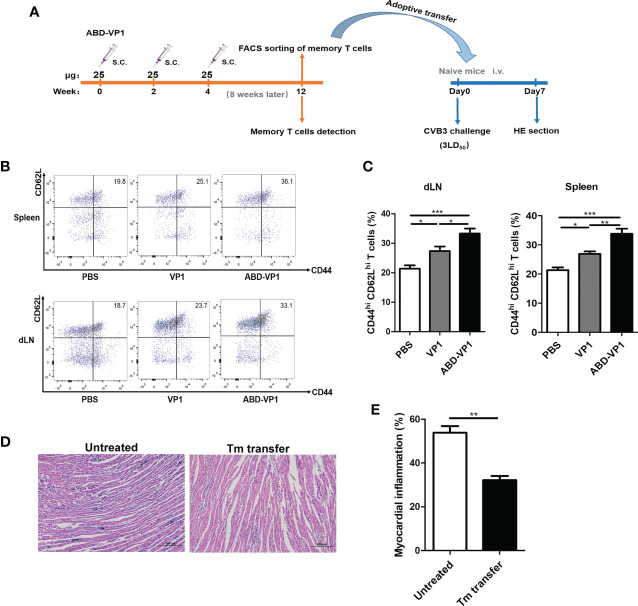
Adoptive transfer of memory T cells derived from ABD-VP1-immunized mice protected naive mice against *CVB3* challenge. **(A)** Timeline of memory T cell detection and transfer. **(B)** Percentages of CD44^hi^ CD62^hi^ memory T cells in spleens and dLNs of immunized mice were detected 8 weeks after the final immunization. **(C)** Statistical analysis of **(B)**. **(D)** Naive mice were adoptively transferred with memory T cells derived from ABD-VP1-immunized mice at the day of *CVB3* (3LD_50_) infection, and the heart histomorphological changes were observed 7 days later. **(E)** Statistical analysis of **(D)**. Each group contained 6 mice. *P < 0.05; **P < 0.01; ***P < 0.001.

Memory T cells play a central, initiating role in recall immune responses to infection (29). In this study, we also determined the production of memory T cells in immunized mice according to the timeline shown in **Figure 6A**. As shown in **Figures 6B, C** the percentages of CD44^hi^ CD62L^hi^ memory T cells in the spleens and dLNs of ABD-VP1 immunized mice were significantly higher than those in VP1-immunized mice. And more importantly, adoptive transfer of these memory T cells derived from the ABD-VP1 group could obviously protect naive mice against *CVB3*-induce viral myocarditis, evidenced by the milder myocardial inflammation and injury (**Figures 6D, E**), confirming the potential immunoprotection effect of ABD-VP1-elicited memory T cells.

## Discussion

There are several strategies for design and development of virus vaccines: using the whole virus (live attenuated vaccine, inactivated vaccine, etc.) or genetically engineered antigens that delivered through different formats (such as recombinant protein vaccines, nucleic acid vaccines or whole cell vaccines). At present, virus vaccines with good immunogenicity and entering clinical trials (such as influenza vaccines and coronavirus vaccines) are mostly whole virus vaccines and subunit recombinant vaccines (30). Compared with the whole virus vaccines, subunit protein vaccines can better ensure the biological safety due to lacking infectious whole pathogens or genetic components. While these characteristics also determined their relatively low immunogenicity and immunoprotection that need to be further improved. Therefore, various delivery and adjuvant strategies have emerged.

Draining lymph nodes are rich in lymphocytes and antigen presenting cells. They are not only responsible for the antigen collection from peripheral tissues, but also function as important inductive sites for adaptive immune responses (31). The immune microenvironments of dLNs are extremely critical for vaccine-induced immune responses (32). Furthermore, lymphatic circulation could significantly increase the exposure of antigen-loaded antigen presenting cells to antigen-specific lymphocytes (33). Of note, in some viral infections (such as *CVB3* infection), lymphocytes are also important target cells (34). This means lymph nodes also represent the important target organs and virus reservoirs, and the potential virus pool in lymphocytes has also been proven to reduce the success rate of HIV treatment (35). Therefore, the dLNs-targeting strategies are expected to better control virus infection (36), as they could not only effectively induce systemic immune responses, but also could effectively clear the local virus pool in lymph nodes.

Many attempts have been made to deliver vaccines to the dLNs, such as changing molecular weights (MW) of drugs or proteins (37), reducing vascular system permeability (38), or introducing guiding peptides (39) etc. In this study, we applied a streptococcus G protein-derived ABD peptide and hoped that it can facilitate the dLNs-targeting of VP1 vaccine by its non-covalent binding with the serum albumin.

Albumin is a protein abundant in serum, while 20%-30% of them can cross the capillary endothelial barriers and enter the lymphatic system (40). More importantly, with a MW of 66 kDa, albumin exceeds the MW cut-off (45 kDa) that passes through the tight junctions of endothelial cells and the basement membranes of blood vessels (41, 42). Therefore, binding to albumin can significantly facilitate antigen enrichment in dLNs (43). In this study, we chose ABD as the dLNs-targeting peptide, not only because it exhibited a stronger albumin-binding ability than other albumin-binding motifs, such as PAB (peptostreptococcal albumin binding) of the anaerobic Finegoldia magna (44), but also because it showed broader species specificity (humans, monkeys, and mice) and biocompatibility. Moreover, ABD motif has been widely used in therapeutic peptide or protein drugs due to its small volume, stable structure and high solubility (45).

As expected, we did observe a much earlier, more abundant and more persistent enrichment of ABD-VP1 vaccine in the dLNs compared with VP1 vaccine (**Figure 2A**). Similar successful dLNs-targeting was also evidenced in other ABD-fused proteins (46). By fusing ABD with elastin iTEP, a dLN targeting system has been constructed and proven to be able to effectively deliver its vaccine antigen to dLNs, with significantly higher enrichment effect than that of the iTEP system alone (47).

Another important consideration for introducing ABD is to extend the *in vivo* retention time of VP1 vaccine. It is well known that limited biological activities of small proteins are partially attributed to their rapid clearance from bodies. The *in vivo* half-lives of proteins or peptides are mainly determined by MWs and renal filtration. The permeability threshold of kidney glomerular membranes is about 70 kDa, so the clearance rates of proteins with MW>70 kDa are significantly reduced compared with smaller proteins. Wei J et al. (48) reported that the *in vivo* half-life of a 50-70 kDa recombinant protein was only 10-30 minutes in mice and a few hours in humans. In our study, the MW of VP1 protein was about 37 kDa, and its *in vivo* half-life was only less than 15 min. When we introduced ABD peptide, the MW of ABD-VP1 was about 42 kDa, considering that it would combine with serum albumin, the MW of the complex would finally achieve 108 kDa, exceeding the permeability threshold of the kidney glomerular membrane. Accordingly, the serum half-life of ABD-VP1 protein prolonged to about 280 min, increasing about 20 folds. Consistently, previous study also reported that fusion of ABD peptide with Fab segments could prolong the antibody’s residence time in the body by several times (49).

We also noticed that the recruitment and maturation of DCs in the dLNs were obviously increased in ABD-VP1 group. Previous studies reported that the binding of ABD-fused proteins with the serum albumin could not directly promote the antigen uptake by DCs, but it can effectively facilitate the intracellular antigen accumulation by increasing the antigen complex stability in endosomal lysosomes, promote the antigen processing and presentation by DCs, and finally induce powerful CTL responses (11). Herein, greater abundance of IFN*-γ^+^* CD8*^+^* cells were evidenced in both dLNs and spleens of ABD-VP1-immunized mice. Accordingly, much alleviated viral myocarditis was also evidenced in the ABD-VP1 group, reflected by the higher survival rate, limited myocardial inflammation and damage, and the better systolic function of the left ventricle after *CVB3* challenge. More importantly, in this study, we observed that the ABD-VP1 could elicit much more memory T cells, and adoptive transfer of these memory T cells could efficiently protect mice from *CVB3* infection.

Actually, this ABD-mediated dLNs-targeting strategy might not only be applied in preventive vaccines, but also in therapeutic vaccines such as tumor vaccines. It has been proven that dLNs-targeting therapeutic tumor vaccines can not only significantly increase the CD8^+^ T cell immune response induced by tumor vaccines, potentially inhibit tumor growth (50), but also help to effectively eliminate metastatic tumor cells in lymph nodes, significantly inhibiting tumor spread (51, 52). For some protein and peptide vaccines and drugs with short half-lives, this strategy also provided a possible solution for prolonging the *in vivo* stability and increasing their immunogenicity. Of course, to further exploit the potential application of this promising strategy, several challenges still need to be addressed, such as how to further increase the antigen uptake efficiency of antigen presenting cells in the dLNs, and how to further reduce the immunogenicity of ABD peptide and so on.

**Figure 7:**
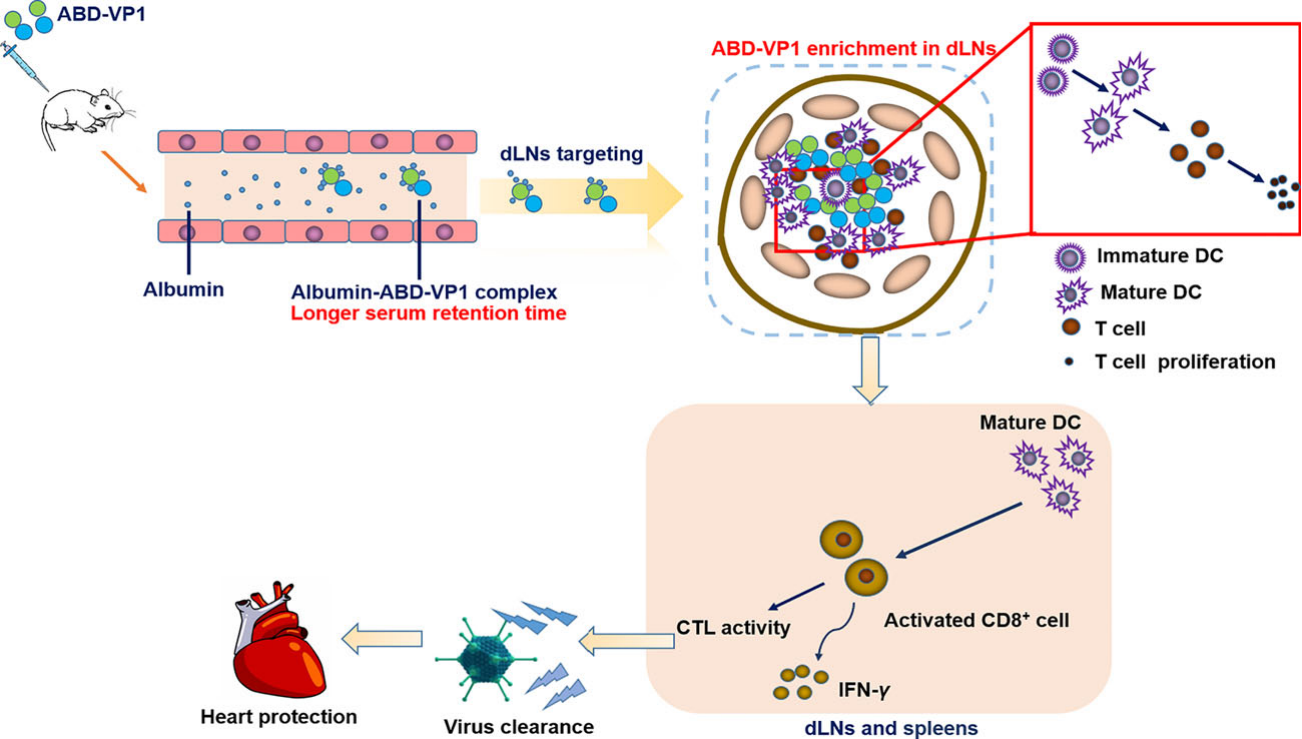
Schematic representation of protection conferred by the dLNs-targeting ABD-VP1 vaccine against *CVB3*-induced viral myocarditis. By forming complexes with albumins, ABD-VP1 vaccine possessed a longer serum half-life and the dLNs-targeting ability. It efficiently recruited and promoted the maturation of DCs, and elicited more robust *CVB3*-specific T cell immune responses as well as a higher level of serum neutralizing antibodies. Accordingly, ABD-VP1 vaccine provided enhanced immunoprotection against *CVB3*-induced myocarditis compared with the original VP1 vaccine.

In this study, we prepared a dLNs-targeting prophylactic vaccine against viral myocarditis by fusing ABD peptide with *CVB3* dominant antigen VP1 protein. We found this novel vaccine could efficiently bind serum albumin *in vitro* and *in vivo*, possessed an obviously prolonged serum half-life. It significantly enriched in the dLNs after subcutaneous immunization and effectively elicited *CVB3*-specific neutralizing antibodies and T cell immune responses, and ultimately provided a more efficient immune protection effect than the simply VP1 vaccine (**Figure 7**).

## Data Availability Statement

The raw data supporting the conclusions of this article will be made available by the authors, without undue reservation.

## Ethics Statement

The animal study was reviewed and approved by Laboratory Animal Ethical Commission of Soochow University.

## Author Contributions

SX conceived of the study. YY designed the experiments. YG performed the experiments. All authors contributed to the article and approved the submitted version.

## Funding

This work was supported by grants from the National Natural Science Foundation of China (31770962, 81970318, 31970844, 31170878, 31370894), A Project Funded by the Priority Academic Program Development of Jiangsu Higher Education Institutions (PAPD), Jiangsu Provincial Innovative Research Team Funding.

## Conflict of Interest

The authors declare that the research was conducted in the absence of any commercial or financial relationships that could be construed as a potential conflict of interest.

## Publisher’s Note

All claims expressed in this article are solely those of the authors and do not necessarily represent those of their affiliated organizations, or those of the publisher, the editors and the reviewers. Any product that may be evaluated in this article, or claim that may be made by its manufacturer, is not guaranteed or endorsed by the publisher.
